# Triple-Negative Breast Cancer Systemic Treatment: Disruptive Early-Stage Developments for Overcoming Stagnation in the Advanced Pipeline

**DOI:** 10.3390/cancers17040633

**Published:** 2025-02-13

**Authors:** Carlos Alonso-Ron, Andrea Vethencourt, Eva González-Suárez, Roke Iñaki Oruezabal

**Affiliations:** 1 Spanish National Cancer Research Center (CNIO), 28029 Madrid, Spain; rioruezabal@cnio.es; 2 Bellvitge Biomedical Research Institute (IDIBELL), 08908 Barcelona, Spain; acvethencourt@iconcologia.net; 3 Catalan Institute of Oncology, 08908 Barcelona, Spain; 4 Faculty of Medicine and Health Sciences, University of Barcelona, 08907 Barcelona, Spain

**Keywords:** triple-negative breast cancer, systemic treatment, targeted therapy, onco-immunotherapy, monoclonal antibody, small molecule inhibitor

## Abstract

New breast cancer (BC) diagnoses will soon reach 2.5–3 million/year worldwide, with 15–25% of them being triple-negative breast cancer (TNBC), the most aggressive type, characterized for lacking the main pharmacological targets: estrogen and progesterone receptors (ERs and PRs), as well as HER2 overexpression. Therefore, chemotherapy remains the almost-unique systemic treatment for TNBC. However, some targeted therapies are recommended for use in combination with chemotherapy; namely, PARP inhibitors for BRCA-mutated TNBC, the immune checkpoint inhibitors pembrolizumab and atezolizumab, as well as the antibody–drug conjugates sacituzumab govitecan and trastuzumab deruxtecan, the latter for HER2low subtypes. Regardless of the limited benefits they provide, other treatments with similar mechanisms of action are being investigated in advanced clinical stages. Further, therapies that benefit other cancers, like PI3K/Akt/mTOR pathway and CDK4/6 inhibitors, are still being investigated for TNBC, although convincing results have not been obtained. Given this scenario, it might appear innovation for TNBC treatments has become stuck. However, the huge unmet medical need drives intense research into the biology of the disease. As a result, emerging disruptive therapies are being tested in early-stage trials, designed for novel targets and applying cutting-edge advances in immunotherapy and precision oncology.

## Simple Summary

Endeavors for addressing the unmet medical needs of triple-negative breast cancer (TNBC) patients are allowing the medical community to understand its biological heterogeneity. Therefore, targeted therapies are being tested together with chemotherapy for systemic treatment. However, these therapies do not appear to provide any drastic curative benefits. Furthermore, new developments positioned in more advanced stages close to approval seem to insist on these same targets. Here, this scenario is compared with the one outlined by the new, more disruptive alternatives of early-stage developments, revealing the need to investigate and validate new therapeutic targets, as well as to constantly assimilate cutting-edge advances in precision oncology and immunotherapy.

## 1. Introduction

The current BC classification contemplates four subtypes: Luminal A-like, Luminal B-like, HER2+, and TNBC. The latter is defined by the absence of ERs and PRs and the lack of HER2 overexpression [1,2]. This nuance emphasizing a ‘lack of overexpression’ rather than an ‘absence of HER2’ is becoming significant, as around 50% of cases previously considered HER2-BC fall now into the ‘HER2low’ category, including many TNBC cases [3,4].

TNBC is considered a heterogeneous entity of different subtypes according to specific biological features. The Lehmann classification has been reviewed after a better understanding of the immune-related tumor microenvironment and now includes the Basal-Like 1 and 2 (BSL 1 and 2), Mesenchymal (M), and Luminal Androgen Receptor (LAR) subtypes. Although it is not yet routinely implemented in clinical practice, it is gaining interest due to its potential to inform tailored therapeutic approaches [5,6,7,8,9]. Regardless, the main treatment is still surgery/radiotherapy (RT) plus chemotherapy (ChT) for early TNBC (eTNBC) and ChT for metastatic disease (mTNBC) [10,11,12]. Several targeted therapies, including immune checkpoint inhibitors (ICIs), are currently employed both in clinical practice and in an experimental context according to biomarkers which are often differentially identified among Lehmann subtypes [4,8,13].

It is estimated that TNBC accounts for 15–25% of all BC, including newly diagnosed cases, being more prevalent in younger women [1,2,14,15,16]. Compared with the HR+ and HER2-overexpressing tumors, TNBC is the most aggressive type, with patients being more predisposed to recurrence and metastasis, with a life expectancy of ten years or less after diagnosis [17,18]. The frequency of recurrence by five years is 30–50% in the operable setting [19,20], with around 45% of patients having distant metastases [21]. In the inoperable setting, the overall survival rate is low, with a median survival time shorter than two years [22,23].

The reported economic burden of TNBC has been reviewed, estimating a mean per-patient annual direct medical costs of 20–100 K USD for the operable setting and 100–300 K for the inoperable one. Systemic therapy costs are only a small portion, the emergency visits and hospitalizations of mTNBC patients being the main drivers [24]. On the other hand, the cost-effectiveness balances of recently approved targeted therapies have been also reviewed, concluding that, whereas ICIs and PARP inhibitors have shown cost-effectiveness in many settings, ADCs are often deemed less cost-effective due to their high prices [25]. However, it should be pointed out that most of the reviewed studies rely on data gathered from pivotal studies. In order to draw more robust conclusions about the cost-effectiveness of these therapies, data from their market launch and implementation in clinical practice are needed. Due to the recent approval of these therapies, to our knowledge, there are still no post-marketing cost-effectiveness studies in the literature.

Seeing that the development of new treatments for TNBC remains a great challenge, here we aim to review the state-of-the-art of systemic treatments, with special attention to targeted and advanced therapies, as well as discussing the upcoming trends. The information is organized according to the regulatory status, mainly in Europe, and the degree of development.

## 2. The Current Systemic Treatment Landscape

### 2.1. Standard of Care Overview

The standard of care for TNBC relies on the recommendations collected in the guidelines of the main medical oncology societies. In general, eTNBC management is surgery plus RT with a (neo)adjuvant systemic therapy of ChT with or without a targeted therapy. Most guidelines recommend ChT for tumors > 0.5 cm or with nodal involvement, regardless of size. While adjuvant and neoadjuvant therapies offer similar survival outcomes, the higher pathological complete response (pCR) rates and surgical benefits of neoadjuvant therapy make it the preferred approach. On the side of mTNBC, both de novo diagnosed or after eTNBC treatment, treatment has a systemic approach [4,8,10,11,12,14,26,27,28]. In this review, we will focus on systemic therapy rather than local treatments.

As the main targetable proteins in BC are lacking in TNBC, its systemic treatment has relied on ChT. The regime backbone had been anthracycline plus cyclophosphamide followed or preceded by a taxane for several years, to which platinum agents—especially carboplatin—and capecitabine began to be added. Later on, some ChT agents in monotherapy were added. Additionally, a better understanding of the molecular footprint of the cancer cells and the tumor microenvironment has led PARP inhibitors, immunotherapy by ICIs, anti-angiogenic therapy, and recently two ADCs to become viable options, especially for mTNBC. As for the former, we refer to olaparib and talazoparib, approved for BC without HER2 overexpression carrying germline BRCA1/2 mutations (gBRCA1/2m), including TNBC cases with this feature, which accounts for 10–20% of all TNBC. As for the ICIs, these are pembrolizumab and atezolizumab, which are specifically approved for TNBC. The anti-angiogenic mAb bevacizumab is approved for metastatic BC (mBC) and recommended for mTNBC in a specific scenario. Regarding the ADCs, they are sacituzumab govitecan, specifically approved for mTNBC, and trastuzumab deruxtecan, approved for mBC HER2low, including mTNBC cases [4,8,10,11,12,13,27,28].

The characterization of PDL1 status is not usually required for eTNBC, as benefits are observed upon neoadjuvant pembrolizumab regardless of it. Therefore, the current standard of care for eTNBC is neoadjuvant ChT (NACT) with an anthracycline–cyclophosphamide regimen followed by taxanes, combined with pembrolizumab. This approach excludes certain special histological subtypes without nodal involvement (e.g., secretory or adenoid cystic carcinomas) or tumors with very low risk, unless there are significant risk factors for excessive immune toxicity associated with ICIs. NACT plus pembrolizumab should be followed by pembrolizumab alone during the adjuvant phase, regardless of whether a pathological complete response (pCR) is achieved. Patients require close monitoring for immune-related adverse events. However, ESMO recommends, if possible, gBRCA1/2 characterization in eTNBC, to be able to offer patients olaparib as an adjuvant if mutations are confirmed. Therefore, there would be two categories of disease as per this theragnostic marker: eTNBC gBRCA1/2m and eTNBC gBRCA1/2wt. Then, for patients with residual disease and gBRCA1/2m, adjuvant therapy with olaparib is recommended. If ICIs were not included in prior treatment, adjuvant capecitabine should be offered to patients with residual disease. The combination of olaparib or ICIs with capecitabine should be avoided and decisions regarding adjuvant therapy should be individualized based on the treating oncologist’s judgment [4,8,10,11,13,26,27,28].

The mTNBC scenario is more intricate since theragnostic marker characterization is recommended, and frequently ChT is accompanied by a targeted therapy. Thus, there are four types of disease: mTNBC PDL1+ gBRCA1/2wt, mTNBC PDL1+ gBRCA1/2m, mTNBC PDL1− gBRCA1/2wt, mTNBC PDL1− gBRCA1/2m. For mTNBC PDL1+, ChT is combined with an ICI. In Europe, the ChT agent used with atezolizumab is the modified taxane nab-paclitaxel. Regarding pembrolizumab, there are more combination options. For those PDL1+ cases in which an ICI is not an option, or for those who are PDL1−, but where gBRCA1/2m is present, the use of a PARP inhibitor in monotherapy (olaparib or talazoparib) is recommended versus ChT. However, if these inhibitors are not available, carboplatin is recommended over taxanes for ChT. Finally, for mTNBC PDL1− gBRCA1/2wt, the recommendation is to check for imminent organ failure. If this risk does not exist, then ChT is recommended with the agent in monotherapy, which can be anthracycline or taxane. On the contrary, if this risk exists, backbone anthracycline–cyclophosphamide–taxane is used, or combining bevacizumab with a taxane. In the case of 3rd line mTNBC treatment, other ChT agents not used before can be used, including vinca alkaloids, such as vinorelbine, or eribulin [4,8,10,12,13,26].

The guidelines also recommend, when physicians find it appropriate, to include patients in clinical trials (CTs) of therapies under investigation, where participation criteria meet their tumor characteristics. Those therapies may be already approved for other indications, including BC, but still be under investigation for TNBC, or even not approved at all for any indication as they are investigational new drugs (INDs).

### 2.2. Chemotherapy Agents

Although the anthracycline–cyclophosphamide plus taxane backbone is still widely used, the current range of agents of choice is quite broad and specific, according to the stage and theragnostic marker status of the disease and the physician’s criteria.

The most employed anthracyclines are doxorubicin and epirubicin, both evolved from daunomycin, although epirubicin has a slightly better cardiovascular safety profile. On the side of taxanes, the agent that has been most widely employed is paclitaxel (taxol), whose safety profile was improved by formulating it wrapped in albumin nanoparticles, giving rise to nab-paclitaxel, which is becoming a preferred choice for TNBC. Platinum-based agents are still under investigation for TNBC; however, carboplatin is currently the preferred choice [4,8,10,11,12,13,26,27,28,29].

### 2.3. Current Use of TNBC Biological Subtyping

Apart from the use of the theragnostic markers PDL1 and gBRCA1/2m, the routine use of TNBC subtyping systems has not yet been established. However, despite Lehman classification not being widely considered in clinical practice and even only slightly mentioned in some guidelines, it suggests each subtype may respond differently to various treatments and it might influence decision-making in certain settings. For instance, the BL1subtype is typically associated with higher proliferation rates and enriched in genes related to cell cycle regulation and DNA damage response, frequently harboring gBRCA1/2m. Therefore, it may respond better to platinum-based ChT and PARP inhibitors. The BL2 subtype is enhanced with growth factor signaling pathways and less responsive to ChT, so targeted therapies for those factors may be chosen. Regarding the M subtype, it is associated with epithelial–mesenchymal transition (EMT) markers, so it might have unique vulnerabilities to therapies targeting, for instance, TGF-β. The LAR subtype exhibits the expression of androgen receptors (ARs), suggesting a potential role for AR hormonal and targeted therapies, a treatment avenue currently under investigation [5,8,12,13,26].

It should be considered that there are several other features found in some cases of TNBC which do not correlate with any Lehman subtype, such as the alteration of the PI3K/Akt/mTOR pathway, one of the most frequently altered in cancer and all TNBC types (~50%), or the less common alteration of the MAPK pathway or CDK4/6 (~3%) [4,7,8,13,16,26].

### 2.4. Targeted Therapies Specifically Approved for TNBC Indication

Only three targeted therapies are specifically approved for TNBC indication by the EMA so far, all being humanized mAbs (Table 1a). The ADC sacituzumab govitecan targets Trop2, a transmembrane glycoprotein highly expressed in several solid tumors, including TNBC. The other two are both ICIs: pembrolizumab, blocking PD1, expressed mainly by T and B cells, and atezolizumab, blocking PDL1, expressed by tumor cells and other immune cells.

### 2.5. Targeted Therapies Approved for BC and Recommended for TNBC

Although not specifically approved for TNBC, the BC indications for which they are approved encompass specific cases of it (Table 1b). Olaparib is approved in Europe for early and metastatic diseases, whereas talazoparib is only approved for metastatic disease. Another one is bevacizumab.

Recently, trastuzumab deruxtecan (T-DXd) was approved by the EMA and FDA for HER2low BC after the Destiny-BREAST04 study [30]. This new entity has arisen recently, defined by harboring a low-level expression of the HER2 protein detectable by IHC, yet with no detectable ErbB2 gene amplification by ISH [3,31]. However, currently available guidelines have not yet fully adopted this definition [10,11,12], indicating that clinical practice is adopting it. Interestingly, this HER2 little expression is sufficient for T-DXd to exert its action, as the small amount of the receptor seems to be enough to serve as an anchoring point for the ADC to release the cytotoxic drug [3,4,8,26,31,32,33]. Although not expressly included in any treatment algorithm for HER2low BC, following the DESTINY-Breast01 results, the ESMO mBC guideline already suggests that T-DXd may have some activity in this indication [12]. Further, ESMO already refers to this non-curative use on its website [34]. Since it is estimated that 40–50% of TNBC cases may fall into HER2low indication [3,31,33], T-DXd has been used in clinical practice in some regions of Europe for HER2low mTNBC patients during the last four years [35].

## 3. TNBC Pipeline

### 3.1. Trends Overview

On one hand, there are medicines approved for other oncology indications, including certain types of BC, seeking approval for TNBC. This categorization is important since, although not a rule of thumb, the fact that a therapy has approval for an indication can accelerate the approval for a new one, even more so if this new indication is somewhat ‘similar’ to the first. On the other hand, there are medicines not yet approved and still being investigated for various indications, including TNBC. Although the advanced-stage pipeline may indicate some lack of innovation with new targets and therapeutic strategies, a brief look at the early pipeline can verify that TNBC is an indication for which very intense R&D is being performed, due to the big medical unmet need.

#### 3.1.1. ADCs

This approach is one of the most promising oncologic systemic treatments, since, although it may imply different mechanisms, it only consists of the local delivery of ChT agents, increasing its efficacy and lowering its toxicity. Several have already been approved and many others are in very advanced stages for different conditions, including BC [32]. The approval of sacituzumab govitecan may have set a precedent for the validation of the TNBC indication of ADC approach and Trop2 as a target, as others are being investigated. In addition, the discovery of T-DXd MoA on HER2low BC is driving diagnostic and treatment paradigms of HER2low TNBC to evolve rapidly and other similar ADCs are being assayed [3,4,8,26,31,32,33].

#### 3.1.2. ICIs

ICIs are a consolidated therapeutic option, even though further knowledge about responsiveness or toxicity is required. However, certain types of cancer are particularly responsive to ICIs because they are highly immunogenic (or ‘hot’), and this is the case for TNBC, while other BC subtypes are not [8,16,26]. Thus, many ICIs are being actively tested in trials including TNBC patients [36,37]. In addition, there are molecules investigated for TNBC for which, although not being ICIs sensu stricto, their MoA relies partially on immune checkpoint inhibition.

#### 3.1.3. PARP Inhibitors

Due to the recommendations of guidelines, it is understandable that PARP inhibitors may be the most frequently found experimental therapy in TNBC as developers seem to be competing to get the best-in-class molecule with this MoA.

#### 3.1.4. Anti-Angiogenic Therapies and PI3K/Akt/mTOR Inhibitors

Many of these are currently being tested, mainly combined with ICIs [8,10,12,26]. It should be pointed out that PI3K/Akt/mTOR inhibitors can, in addition to exerting their action on a pathway that may be pathologically altered in 50% of TNBC, be also anti-angiogenic, since this route is implied in VEGF synthesis or downstream from its receptor [8,13,26,38]. Other anti-angiogenic molecules are tyrosine kinase inhibitors (TKI) with different levels of affinity for any of the VEGFR subtypes, or even a mAb anti-VEGFR2.

#### 3.1.5. CDK4/6 Inhibitors

Approved for ER+ HER2-BC some time ago, although conclusive results have not yet been obtained regarding their benefit in TNBC. They are still being tested.

#### 3.1.6. MEK Inhibitors

Although the MAPK pathway is not frequently altered in TNBC, some of MEK inhibitors are being investigated [8,13,26].

#### 3.1.7. EGFR-Targeting Therapies

As TNBC may also express EGFR, some molecules targeting it are being investigated for TNBC, mostly in early stages [8,13,26].

#### 3.1.8. Hormonal and Antihormonal Therapies

Although these are not targeted therapies sensu stricto [39], they should be mentioned as a trend. This might at first seem paradoxical for a type of BC that is defined by the absence of ERs and PRs. However, it must be considered that TNBC can present expression of other types of HRs. Therefore, AR antagonists and modulators are actively tested in the LAR subtype, which accounts for approximately 20–40% of all TNBCs [8,26]. There is also a line investigating the benefits of estradiol and its analogs in TNBC expressing the ER-β. It has been observed that in this sort of TNBC estradiol has an antiproliferative and antimetastatic effect, and there are even some CTs in phase II (e.g., NCT03941730) studying the possibilities of this treatment [40].

#### 3.1.9. Disruptive Approaches in Early-Stage Cases

This category includes medicines intended for novel targets in TNBC and cutting-edge onco-immunotherapy [8,26].

### 3.2. Targeted Therapies Approved for BC and Under Investigation for TNBC

All of these therapies are small molecule inhibitors except the ADC targeting HER2 trastuzumab emtansine [3,4,26,31,32] (Table 2). The PI3K/Akt/mTOR inhibitors are actively studied; regardless, there is still little evidence for their benefit: this includes the mTORC1 inhibitor everolimus, the PI3Kα-selective inhibitor alpelisib, and the Akt inhibitor capivasertib. This includes the CDK4/6 inhibitors ribociclib, palbociclib, and abemaciclib, as well as two inhibitors of the ErbB family receptors, lapatinib, with an affinity for both EGFR and HER2, and neratinib, with broader affinity for EGFR and HER2/3/4 [8,13,16,26].

### 3.3. Targeted Therapies Approved for Other Indications and Under Investigation for TNBC

Details are in Table 3.

#### 3.3.1. mAbs

Almost all of these are ICIs [4,8,13,16,26]. Avelumab and durvalumab are both anti-PDL1 mAbs, whereas nivolumab, cemiplimab, dostarilimab, sintilimab, and retifanlimab are all anti-PD1 mAbs. Additionally, there is tremelimumab, targeting CTLA4. Avelumab and durvalumab are the ones more developed for TNBC indication. The list also includes cetuximab, a mAb approved a long time ago which binds EGFR.

Denosumab is also included. Although not an ICI sensu stricto, it blocks RANKL, preventing its interaction with RANK, a novel and promising immunogenic target in BC. In both preclinical models and patient samples from the D-BEYOND study (NCT01864798), the inhibition of RANK signaling by denosumab has enhanced its immunogenicity. While denosumab is already approved for osteoporosis and cancer-related bone loss, its potential as a targeted therapy for BC, particularly in TNBC, is still in early development. RANK is expressed in approximately 35% of TNBC tumors and is associated with poor prognosis. The recently completed GeparX phase II (NCT02682693) study has explored the benefits of denosumab for neoadjuvant regimes in HER2-BC patients including TNBC. The ongoing D-BIOMARK trial, a hypothesis-generating study, is exploring the immune activation of the tumor microenvironment with denosumab in HER2 BC, including TNBC [41,42,43,44].

#### 3.3.2. Small Molecule Inhibitors

This list is larger and, interestingly, the only molecule in phase III is the PARP inhibitor niraparib.

There are three anti-angiogenic VEGFR inhibitors. One of them is lenvatinib, a molecule with a high affinity for VEGFR, but also for FGFR and PDGFR, which may contribute to its anti-angiogenic action. Additionally, its benefits might be due to the direct effect over FGFR itself, as it is usually overexpressed in TNBC tumor cells [4,8,26,38]. The others are fruquintinib, specific for VEGFR with no significant discrimination between receptor subtypes [45], and cabozantinib, an inhibitor with affinity for the tyrosine kinase domain of several receptors, including VEGFR2 [45]. Then, there are three MAPKK/MEK inhibitors, binimetinib, selumetinib, and trametinib [8,13,16,26], and two EGFR inhibitors, erlotinib and gefitinib [13,26].

Listed also are two other inhibitors of novel and interesting targets for TNBC. One of them is crizotinib, which interacts with the aberrant receptors ALK and ROS1 and is approved for conditions harboring these mutations. There is an active phase II trial, with a basket design, for patients with ‘lobular TNBC’, one ‘rare subtype’ that accounts for 5–10% of all cases [46]. They are checked for mutations in E-Cadherin since an effect of crizotinib had been seen in BCs with defects in this protein [47]. The other molecule is the first-in-class selinexor, a Selective Inhibitor of Nuclear Export (SINE), which binds the nuclear membrane protein XPO1, inhibiting oncogene transcripts’ export [48].

#### 3.3.3. Other Therapies

These therapies are not considered strictly as targeted therapies [39]: the oncolytic virus talimogene laherparepvec (or T-VEC) [8,26] and the AR antagonists bicalutamide and enzalutamide [26].

### 3.4. Investigational Targeted Therapies in the Advanced Clinical Development Stage for TNBC

This category includes medicines not yet approved and under investigation in phase III for several indications including TNBC.

#### 3.4.1. mAbs 

**Figure 1:**
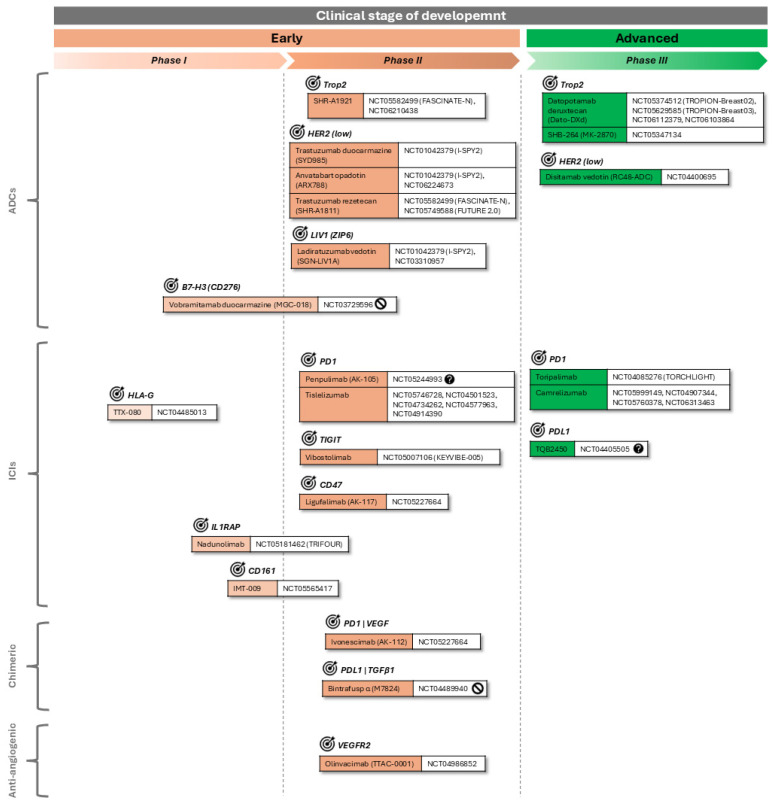
Investigational targeted therapies for TNBC: mAbs. Experimental mAb-based targeted therapies under investigation for TNBC indication in different clinical development stages are represented. Ongoing CTs at highest phase for each IND are shown. ‘Ongoing’ refers to the statuses of “not yet recruiting”, “recruiting”, and “active, not recruiting” as stated by August 2024 in the National Library of Medicine USA (NLM) registry Clinicaltrials.gov. In some cases, no ongoing CTs were found, so the last completed terminated CTs at highest phase are shown instead, labeled with 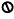. Additionally, the 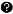 label is used for those studies with an unknown status. Targets of each mAb are indicated and labeled with the symbol 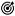. Left panel specifies the type of mAb: “ADC”, antibody–drug conjugate; “ICI”, immune checkpoint inhibitor; “chimeric” refers to molecules that, although built on them, cannot be considered mAbs sensu stricto.

Details are in Figure 1.

Several ADCs targeting Trop2 are being developed for TNBC, among other indications [49]. One of them is datopotamab deruxtecan, characterized for carrying as a conjugated drug deruxtecan, which is a topoisomerase I inhibitor 10 times more potent than SN-38. SKB264 is another one, also conjugated with a topoisomerase I inhibitor, this time belotecan. Further, there is the ADC targeting HER2, disitamab vedotin, built on the mAb hertuzumab and conjugated with MMAE, a microtubule inhibitor [3,4,8,26,31].

Additionally, there are two anti-PD1 ICIs: toripalimab and camrelizumab [8,26].

#### 3.4.2. Small Molecule Inhibitors

**Figure 2:**
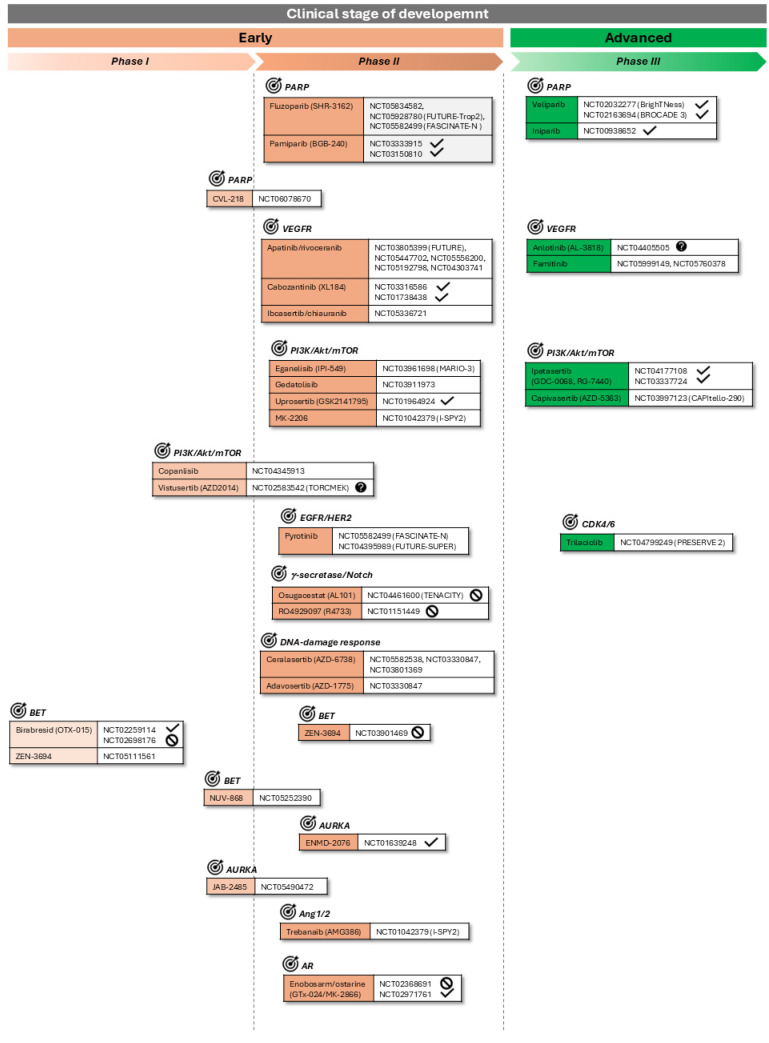
Investigational targeted therapies for TNBC: small molecule inhibitors. Experimental small molecule inhibitors under investigation for TNBC indication in different clinical development stages are represented. Ongoing CTs at the highest phase for each IND are shown. ‘Ongoing’ refers to the statuses of “not yet recruiting”, “recruiting”, and “active, not recruiting” as stated by August 2024 in the National Library of Medicine USA (NLM) registry Clinicaltrials.gov. In some cases, no ongoing CTs were found, so the last completed, labeled with 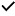, or terminated, labeled with 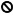, CTs at the highest phase are shown instead. Additionally, the 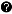 label is used for those studies with an unknown status. Targets of each molecule are indicated and labeled with the symbol 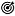.

Details are in Figure 2.

Regarding PARP inhibitors, there are two molecules with promising results after the completion of phase III: veliparib [4,8,26,50] and iniparib [10]. In addition, there are two anti-angiogenic TKIs: anlotinib (or AL-3818), which targets both VEFGR and FGFR, and famitinib, which also targets VEGFR and FGFR, together with several other kinases [8,26]. Then, there are two interesting molecules targeting the PI3K/Akt/mTOR pathway, both of them Akt inhibitors: ipatasertib and capivasertib [8,26]. Finally, there is the CDK4/6 inhibitor trilaciclib [26].

### 3.5. Investigational Targeted Therapies in Early Clinical Development Stage for TNBC

This category includes INDs in phase I to II. In many cases, they target novel and promising proteins from a therapeutic perspective. Several of them are being tested in phase II in the adaptive I-SPY2 study and the umbrella-designed FASCINATE-N [51].

#### 3.5.1. mAbs 

Details are in Figure 1.

Regarding ADCs, there is the anti-Trop2 SHR-A1921. Then, there are up to three intended for HER2low TNBC: trastuzumab duocarmazine (SYD985), trastuzumab rezetecan (SHR-A1811), and anvatabart opadotin. Two of them are built on trastuzumab but conjugated with different molecules: SYD985 carries the alkylating agent duocarmicin, whereas SHR-A1811 carries the topoisomerase I inhibitor SHR9265. Anvatabart opadotin is built on a different anti-HER2 mAb and is conjugated with the microtubule inhibitor MMAF.

Another two ADCs target novel surface proteins. Ladiratuzumab vedotin binds LIV1—a zinc transporter—for the local delivery of MMAE [8,26,52]. The other one, vobramitamab duocarmazin, applies an interesting strategy: it binds B7-H3, a checkpoint molecule expressed by tumor cells with other additional nonimmunologic pro-tumorigenic functions [36], and is conjugated with duocarmicin. Therefore, it has several MoAs: ICI, blocking tumorigenic signals, and the localized discharge of an alkylating agent.

Regarding ICIs, there are two anti-PD1 options: penpulimab and tislelizumab [8]. There are other mAbs targeting modulators of the tumor immune environment, but different from the more established ones, such as PD1, PDL1, or CTLA4 [37]. One example is ligufalimab, targeting CD47, which works somewhat as a checkpoint inhibitor providing ‘don’t eat me’ signals to macrophages. CD47 has been already described as a promising target for cancer in general and TNBC in particular in preclinical models [37,53,54]. Another one is vibostolimab, which binds the immune checkpoint TIGIT, whose ligands are CD112 and CD155 of T-lymphocytes that destroy tumor cells [55]. Further, there is IMNT-009, which targets CD161, a receptor expressed by different immune cell types [56], and nadunolimab, which might be not considered an ICI sensu stricto since it does not block any immune checkpoint molecule. However, nadunolimab is an immunomodulating mAb since it binds IL1RAP, blocking the IL-1α/1β signaling involved in both tumorigenesis and ChT resistance [57]. Finally, there is TTX-080, which targets HLA-G [58].

Regarding anti-angiogenic mAbs, there is currently a trend in cancer therapy, including BC, to target VEGFR2 [8,26,45], and one candidate, olinvacimab, is currently being assayed in TNBC.

This assessment concludes with two interesting molecules which are not exactly ‘conventional mAbs’. One is ivonescimab, a bispecific antibody binding PD1 and VEGF, functioning therefore simultaneously as an ICI and as an anti-angiogenic therapy. The other one is bintrafusp alfa, also a bifunctional protein: one extreme binds PDL1 as a conventional anti-PDL1 ICI and the other extreme, which is the binding site of TGFβRII, serves as a TGF-β ‘trap’. High levels of HMGA2 expression might relate to the benefits of this treatment for TNBC; however, results are not yet clear [59,60].

#### 3.5.2. Small Molecule Inhibitors of Consolidated Targets 

Details are in Figure 2.

The term ‘consolidated’ refers to targets for which therapies have been used in oncologic treatments for some time.

Three PARP inhibitors are listed: fluzoparib and pamiparib [8] together with CVL-218. Then, there are another three VEGFR inhibitors [8,26]: apatinib (or rivoceranib), which specifically binds VEGFR2; cabozantinib, which targets VEGFR2 as well as several other kinases; and ibcasertib (or chiauranib), which targets VEFGR, PDGFR, and other kinases.

Regarding the PI3K/Akt/mTOR pathway, there are three new PI3K inhibitors [8]. One is copanlisib, a drug not yet fully approved in Europe as it had achieved the EMA’s orphan designation for marginal zone lymphoma but was recently withdrawn by the company [61]. The other two are eganelisib, which is both a PI3K and mTOR inhibitor, and gedatolisib. In addition, there is a mTOR1/2 inhibitor called vistusertib and two Akt inhibitors, uprosertib [8,26] and MK-2206 [33].

To conclude the list, there is the EGFR/HER2 inhibitor pyrotinib [51].

#### 3.5.3. Small Molecule Inhibitors for Novel Targets 

Details are in Figure 2.

The term ‘novel’ refers to targets for which therapies are not yet approved for any indication and are waiting to be validated in a clinical setting. In any case, many of them might represent a breakthrough in oncological therapies soon.

### 3.6. γ-Secretase/Notch Receptor Inhibitors

These represent an upcoming trend that relies on promising preclinical results but still have disappointing clinical outcomes [62]. Nevertheless, two are being tested in TNBC: osugacestat, whose benefits for other indications in PDx models have been recently described [63], and RO4929097 [26,62]. CTs for both have been terminated, corroborating the difficulties encountered in generating clinical evidence of the benefits of this sort of inhibitor for all oncological indications. However, everything suggests that more drugs of this type will continue to be tested and developed [62].

### 3.7. DNA-Damage Response Inhibitors

Besides PARP inhibitors, other alternatives targeting the DNA-damage response are being explored for TNBC. For instance, there is ceralasertib, an ATR inhibitor, and adavosertib, an inhibitor of WEE1 [8].

### 3.8. BET Protein Inhibitors

The therapeutic approach focused on epigenetics is also reaching TNBC and specifically targeting BET proteins, a family of four known members so far, BRD1 to 4. They recognize the acetylated lysine residues of histones and interact with the transcription machinery for gene expression. Therefore, in a pathological context with an aberrant acetylation pattern, they play a highly relevant role in the expression of oncogenic and other disease-associated genes. For these reasons, they have become promising targets, and different small molecule inhibitors are currently being tested for several conditions, BC, and especially TNBC, being one of them. However, some toxicity issues are being encountered [64]. Some examples are birabresid, which binds BRD2, 3, and 4, ZEN-3694, which targets also BRD2/3/4, and NUV-868, this time specific for BRD2.

### 3.9. Aurora Kinase Inhibitors

Aurora kinase family is another promising target under research for the last years, especially members A and B, whose functions are best known. Aurora kinase A, or AURKA, seems to play roles in the most characteristic processes altered in carcinogenesis like cell division, EMT, metastasis, and apoptosis. Accordingly, numerous inhibitors have been developed which are being assayed for several conditions such as BC and specifically TNBC [65]. Among them, perhaps the one that went furthest in its development for TNBC was ENMD-2076 [65]. Additionally, JAB-2485 is also being tested for TNBC, among other cancers.

### 3.10. Anti-Angiogenic

There is an interesting small peptide called trebananib which binds and neutralizes the surface glycoprotein Ang1/2, having therefore an anti-angiogenic activity, and it is considered an oncologic target with high therapeutic potential [33,66].

### 3.11. Other Small Molecules 

Another interesting molecule is enobosarm or ostarine. It is not exactly an inhibitor, but a modulator, targeting ARs. Indeed, it is categorized as an ‘selective androgen receptor modulator (SARM)’, since it can mimic testosterone in men. However, it has a therapeutic value in women, for instance in treating AR+ BC and AR+ TNBC since it can block the use of androgens by the tumor cells [8,26].

## 4. Onco-Immunotherapy 

Other approaches apart from ICIs are also explored for TNBC (see Table 4).

### 4.1. Oncolytic Viruses

This category includes two therapies: pelareorep and BT-001. The MoA of the latter is interesting since it is an oncolytic virus, like T-VEC, providing immunogenicity, and, additionally, it can locally deliver an ICI.

### 4.2. Vaccines

There is a ‘personalized synthetic long peptide vaccine’ [26,67]. There is also a phase I/II CT testing up to six different vaccines, both adenovirus-based or yeast-derived, targeting different neoantigens. Additionally, there is a first-in-human CT testing personalized mRNA-based vaccines [7] and another vaccine called PVX-410 [8].

### 4.3. CAR-Cell Therapy

As in other solid tumors, this is actively being tested in BC and specifically in TNBC, although its efficacy as well as the benefits it can provide for patients and healthcare systems are still to be clarified. For this reason, most of the CTs are still stuck in phase I. As it could not be otherwise, the most studied in TNBC is CAR-T so far, with therapies being designed for different targets, such as ROR1 and mesothelin [68]. MUC1 and its aberrantly glycosylated variant, TnMUC1, is another target against which CAR-Ts were engineered. Additionally, CAR-NK cell therapy was also developed for MUC1 [68].

To conclude, there are two more examples. The first is CARTs targeting NKG2DL, which is the ligand NKG2D receptor, a checkpoint molecule that, in a pathologic context, allows tumor cells to escape NK cell surveillance and its cytotoxic action over them. This way, NKG2DL-CARγδT therapy is being tested for several solid tumors, including TNBC. On the other hand, the efficacy of bivalent CART engineered against both EGFR and the checkpoint molecule B7-H3 is being similarly explored [36,68,69].

## 5. Remarks/Conclusions

For a long time, ChT was the only systemic treatment available for TNBC. While it remains the main systemic therapy approach, some targeted therapies have now been established as options to complement it, or even as monotherapies. These include PARP inhibitors, immunotherapy by PD1/PDL1-blocking ICIs, and ADCs. After our analysis we have been able to verify that advanced-stage developments seem to insist on molecules with the same mechanism of action. There are several PARP inhibitors in phase III/II that appear to be competing to be the best-in-class molecule for TNBC indication, and the same is true for anti-PDL1/PD1 ICIs. Regarding the latter, it is necessary to mention that, although they still have limited use in BC, they have shown efficacy in TNBC, as it is the most immunogenic subtype among BCs. However, the identification of response biomarkers would allow clinicians to avoid overtreatment, as it is crucial not to underestimate the immune-mediated effects of ICIs, since their use in early stages may, at times, subject patients to prolonged and even lifelong side effects, such as endocrinopathies. On the side of the ADCs, a type of therapy which is revolutionizing oncology treatment, the same happens, as there are several molecules in late-stage development targeting Trop2 and HER2 (in this case for HER2low TNBC).

We have also noticed after scanning the advanced TNBC pipeline that there are several drugs already approved for other oncological indications, with which this disease can share therapeutic targets (e.g., PIK3A/Akt/mTOR, CDK4/6, VEGF/VEGFR, EGFR, and AR). However, none of them has yet demonstrated surprising benefits, as several other experimental therapies with these mechanisms of action are also being tested in TNBC.

At the same time, discovering new targets with therapeutic potential leads to the design of new molecules targeting them, which are being tested in early stages of development for different oncological conditions, including TNBC. Among these novel targets are the BET proteins and AURKA, and immunomodulatory molecules such as B7-H3, TIGIT, CD47, or IL1RAP. Some of these molecules might prove a fair efficacy–toxicity balance that will allow their approval and use in the future, although sufficient evidence has not yet been generated to validate their therapeutic potential. The same may happen with cell therapy and other strategies such as oncolytic viruses and vaccines.

However, it seems that the key to developing innovative therapies for TNBC lies in continuing to study its biological heterogeneity in depth, defining biomarkers, and discovering and validating new targets. This will probably go hand in hand with the challenge of implementing new tumor biological classifications and patient stratifications for the design of CTs.

## Figures and Tables

**Table 1:** Targeted therapies currently used for TNBC indication. Although differences between the USA and Europe are subtle and very specific (see main text), notice that the details of the indications specified are those of the EMA. Upper table rows gather those medicines specifically approved for TNBC indication (**a**), whereas lower rows gather those medicines approved for BC and recommended by ESMO guidelines for those TNBC cases that encompass the tumor characteristics for which they are indicated (**b**). General information and the indication details were taken from EMA digital resources. Ongoing trials refer to the statuses of “not yet recruiting”, “recruiting”, and “active, not recruiting” as stated by August 2024 in the National Library of Medicine USA (NLM) registry Clinicaltrials.gov. Details of the participation criteria in these trials are provided.

**(a) Therapies specifically approved for TNBC.**		
**General Information**	**Indication Details (EMA)**	**Ongoing TNBC CT at Highest Phase**
▪ Brand name: Trodelvy^®^ ▪ INN: sacituzumab govitecan ▪ MoA: ADC targeting Trop2	▪ TNBC: unresectable/metastatic disease in monotherapy, pivotal study ASCENT (NCT02574455). ▪ Other: unresectable/metastatic HR+ HER2+ BC.	Phase III. ▪ Locally advanced/mTNBC: ISIdE (NCT05552001) ASCENT-003 (NCT05382299) ASCENT-004 (NCT05382286) ▪ eTNBC with residual disease: ASCENT-005 (NCT05633654) ▪ Primary HER2-BC including TNBC: SASCIA (NCT04595565)
▪ Brand name: Keytruda^®^ ▪ INN: pembrolizumab ▪ MoA: ICI targeting PD1.	▪ TNBC: Unresectable/metastatic disease in combination with ChT, pivotal study KEYNOTE-355 (NCT02819518). Locally advanced/early-stage disease at high risk of recurrence in combination with ChT, pivotal study KEYNOTE-522 (NCT03036488). ▪ Other: melanoma, NSCLC, classical Hodgkin lymphoma, urothelial carcinoma, head and neck squamous cell carcinoma, renal cell carcinoma, MSI-H/dMMR cancers, esophageal carcinoma, endometrial carcinoma, cervical cancer, GEJ adenocarcinoma.	Phase III. ▪ Locally advanced mTNBC: KEYNOTE-522 (NCT03036488) ASCENT-004 (NCT05382286) TROPION-Breast03 (NCT05629585) TROPION-Breast05 (NCT06103864) ▪ eTNBC with residual disease: CAPPA (NCT05973864) ASCENT-005 (NCT05633654) ▪ eTNBC: NordicTrip (NCT04335669) SCARLET (NCT05929768) NCT06393374 NCT05812807 ▪ eTNBC/HR-low HER2-BC. TROPION-Breast04 (NCT06112379)
▪ Brand name: Tecentriq^®^ ▪ INN: atezolizumab ▪ MoA: ICI targeting PDL1	▪ TNBC: unresectable locally advanced/metastatic disease in combination with ChT, pivotal study Impassion130 (NCT02425891) ▪ Other: urothelial carcinoma, NSCLC.	Phase III. ▪ Locally advanced/eTNBC with tested PDL1 status: NCT03281954. ▪ Locally advanced/mTNBC PDL1+: EL1SSAR (NCT04148911) ▪ Relapsing recurrent eTNBC: NCT03371017
**(b) Therapies approved for BC and recommended for TNBC.**		
**General information**	**Indication details (EMA)**	**Ongoing TNBC CT at highest phase**
▪ Brand name: Lynparza^®^ ▪ INN: olaparib ▪ MoA: PARP small molecule inhibitor.	▪ BC HER2- with gBRCA1/2m: Locally advanced/metastatic disease in monotherapy, pivotal study OlimpiAD (NCT02000622) High-risk early disease in monotherapy or in combination with endocrine therapy, pivotal study OlympiA (NCT02032823) ▪ Other: ovarian cancer, adenocarcinoma of the pancreas, prostate cancer.	Phase III. ▪ TNBC/HER2-BC with gBRCA1/2m: PARTNER (NCT03150576) ▪ TNBC or HER2-/low BC: NCT06112379
▪ Brand name: Talzenna^®^ ▪ INN: talazoparib ▪ MoA: PARP small molecule inhibitor.	▪ BC HER2- with gBRCA1/2m: locally advanced/metastatic disease in monotherapy, pivotal study EMBRACA (NCT01945775) ▪ Other: metastatic castration-resistant prostate cancer (mCRPC).	Phase II. ▪ Advanced/mTNBC: NCT03911973 START (NCT05035745) ▪ mBC with a Homologous Recombinant Deficiency (HRD) signature: NCT05288127, ▪ Advanced BC with any HER2 status: I-SPY2 (NCT01042379) ▪ TNBC: PERSEVERE (NCT04849364)
▪ Brand name: Avastin^®^, biosimilars ▪ INN: bevacizumab ▪ MoA: mAb anti-VEGFA	(Referring to Avastin^®^) ▪ Metastatic BC: in combination with ChT, pivotal studies E2100 (NCT00028990) and RIBBON-1 (NCT00262067) ▪ Other: colon/rectum carcinoma; NSCLC; renal cell, epithelial ovarian, fallopian tube, or primary peritoneal cancer; carcinoma of the cervix.	Phase III. ▪ Locally advanced/mTNBC BLIS subtype: NCT05806060
▪ Brand name: Enhertu^®^ ▪ INN: trastuzumab deruxtecan ▪ Other names: T-Dxd, DS-8201, AZD-4552 ▪ MoA: ADC targeting HER2	▪ Unresectable/mBC: HER2+ in monotherapy. HER2-low in monotherapy, pivotal study DESTINY-Breast04 (NCT03734029) ▪ Other: HER2+ NSCLC or gastric/GEJ adenocarcinoma.	Phase II. ▪ mTNBC HER2-low: BEGONIA (NCT03742102) NCT05953168 ▪ Advanced BC with any HER2 status: I-SPY2 (NCT01042379)

**Table 2:** Targeted therapies approved for BC under investigation for TNBC. Although differences between the USA and Europe are minor, notice that the details of the indications specified are those of the EMA. Both types of molecularly targeted therapies, mAb-based and small molecule inhibitor-based, are compiled. Notice only one mAb-based targeted therapy is listed: trastuzumab emtansine. General information and the indication details were taken from EMA digital resources. Ongoing trials refer to the statuses of “not yet recruiting”, “recruiting”, and “active, not recruiting” as stated by August 2024 in the National Library of Medicine USA (NLM) registry Clinicaltrials.gov. Details of the participation criteria in these trials are provided.

General Information	Indication Details (EMA)	Ongoing TNBC CT at Highest Phase
▪ Brand name: Kadcyla^®^ ▪ INN: trastuzumab emtansine ▪ Other names: T-DM1 ▪ MoA: ADC targeting HER2	▪ BC: HER2+ eBC with residual invasive disease in monotherapy. HER2+ mBC in monotherapy.	Phase II. ▪ Advanced BC with any HER2 status: I-SPY2 (NCT01042379)
▪ Brand names: Afinitor^®^, generics ▪ INN: everolimus ▪ MoA: mTORC1 small molecule inhibitor.	(Referring to Afinitor^®^) ▪ BC: HR+ HER2/neu- advanced BC in combination with endocrine therapy. ▪ Other: neuroendocrine tumors of pancreatic, gastrointestinal, or lung origin; renal cell carcinoma.	Phase III. ▪ Locally advanced/mTNBC LAR subtype with PI3K/Akt/mTOR pathway mutation: NCT05954442
▪ Brand name: Piqray^®^ ▪ INN: alpelisib ▪ MoA: PI3Kα small molecule inhibitor.	▪ BC: HR+ HER2- locally advanced/metastatic disease with a PIK3CA mutation in combination with fulvestrant.	Phase III. ▪ Locally advanced/mTNBC with either a PIK3CA mutation or PTEN loss: EPIK-B3 (NCT04251533)
▪ Brand name: Truqap^®^ ▪ INN: capivasertib ▪ MoA: Akt small molecule inhibitor.	▪ BC: ER+ HER2- locally advanced or metastatic disease with PIK3CA/AKT1/PTEN alterations in combination with fulvestrant.	Phase III. ▪ Locally advanced/mTNBC: CAPItello-290 (NCT03997123)
▪ Brand name: Kisqali^®^ ▪ INN: ribociclib ▪ MoA: CDK4/6 small molecule inhibitor.	▪ BC: locally advanced/metastatic HR+ HER2- disease in combination with endocrine therapy.	Phase I/II. ▪ mTNBC AR+: NCT03090165
▪ Brand name: Inbrance^®^ ▪ INN: palbociclib ▪ MoA: CDK4/6 small molecule inhibitor.	▪ BC: locally advanced/metastatic HR+ HER2- disease in combination with endocrine therapy.	Phase II. ▪ Untreated/mTNBC: NCT05067530
▪ Brand name: Verzenio(s)^®^ ▪ INN: abemaciclib ▪ MoA: CDK4/6 small molecule inhibitor.	▪ BC: in combination with an endocrine therapy, for: Node-positive early disease at high risk of recurrence Locally advanced/metastatic HR+ HER2- disease.	Phase II. ▪ Locally advanced/mTNBC AR+: ABBICAR (NCT06365788) ▪ Advanced BC with any HER2 status: I-SPY2 (NCT01042379) ▪ Surgically resectable, chemotherapy-resistant TNBC: NCT03979508
▪ Brand names: Tyverb^®^, Tykerb^®^, generics. ▪ INN: lapatinib ▪ MoA: EGFR and HER2 small molecule inhibitor.	(Referring to Tyverb^®^) ▪ BC overexpressing HER2: Advanced/metastatic disease in combination with ChT. Metastatic disease HR- in combination with trastuzumab. Metastatic disease HR+ in combination with endocrine therapy.	Phase I. ▪ mTNBC with no mutations in BRCA1/2: NCT02158507 (pilot study)
▪ Brand name: Nerlynx^®^ ▪ INN: neratinib ▪ MoA: EGFR and HER2/3/4 small molecule inhibitor.	▪ BC: early HR+ HER2 overexpressed/amplified disease.	Phase III. ▪ HR+ HER2-BC with tBRCA1/2m or TNBC irrespective of BRCA1/2 status: NCT04915755

**Table 3:** Therapies approved for other indications under investigation for TNBC. Although differences between the USA and Europe are minor, notice that the details of the indications specified are those of the EMA. Upper table rows gather mAb-based targeted therapies (**a**), whereas middle rows gather small molecule inhibitor-based ones (**b**). In the lower rows medicines not considered targeted therapies sensu stricto are compiled (**c**). General information and the indication details were taken from the EMA digital resources. Ongoing trials refer to the statuses of “not yet recruiting”, “recruiting”, and “active, not recruiting” as stated by August 2024 in the National Library of Medicine USA (NLM) registry Clinicaltrials.gov. Details of the participation criteria in these trials are provided.

**(a) mAb-Based Targeted Therapies**		
**General Information**	**Indication Details (EMA)**	**Ongoing TNBC CT at Highest Phase**
▪ Brand name: Bavencio^®^ ▪ INN: avelumab ▪ Target: PDL1	Merkel cell, urothelial and renal cell carcinoma.	Phase III. ▪ High-risk TNBC: A-brave (NCT02926196)
▪ Brand name: Imfinzi^®^ ▪ INN: durvalumab ▪ Target: PDL1	NSCLC, SCLC, biliary tract cancer, and hepatocellular carcinoma.	Phase III. ▪ eTNBC with no known BRCA1/2 mutation. TROPION-Breast03 (NCT05629585) ▪ TNBC and HER2low BC: NCT06112379 ▪ Unresectable/mTNBC PDL1+: NCT06103864
▪ Brand name: Opdivo^®^ ▪ INN: nivolumab ▪ Target: PD1	Melanoma; NSCLC; malignant pleural mesothelioma; renal cell carcinoma; classical Hodgkin lymphoma; squamous cell cancer of the head and neck; urothelial carcinoma; dMMR or MSI-H colorectal cancer; oesophageal squamous cell carcinoma; gastric, gastro-oesophageal junction or oesophageal adenocarcinoma.	Phase II. NCT02393794, NCT03487666, NCT02499367, NCT04159818, NCT05888831, NCT04180371, NCT03818685, NCT03449108, NCT04331067, NCT03414684, NCT03546686. In general participation criteria are TNBC or advanced solid tumors including TNBC, and none of the CTs ask for PDL1 status test.
▪ Brand name: Libtayo^®^ ▪ INN: cemiplimab ▪ Target: PD1	Cutaneous squamous cell carcinoma, basal cell carcinoma, NSCLC, and cervical cancer.	Phase II. ▪ Advanced BC with any HER2 status: I-SPY2 (NCT01042379) ▪ Advanced/metastatic cancer including TNBC: NCT04916002 ▪ Locally advanced HER2- or TNBC PDL1/2+: NCT04243616
▪ Brand name: Jemperli^®^ ▪ INN: dostarlimab ▪ Other name: TSR042 ▪ Target: PD1	Endometrial cancer.	Phase II. ▪ Advanced BC with any HER2 status: I-SPY2 (NCT01042379) ▪ Recurrent/mTNBC: NCT04837209
▪ Brand name: Tyvyt^®^ ▪ INN: sintilimab ▪ Target: PD1	Peripheral T-cell lymphoma.	Phase II. ▪ Locally advanced non-metastatic TNBC: NeoSACT (NCT04877821) ▪ Pretreated mTNBC: NCT05386524 ▪ Locally advanced/metastatic solid tumors PDL1+ including TNBC: NCT06078670
▪ Brand name: Zynyz^®^ ▪ INN: retifanlimab ▪ Target: PD1	Metastatic Merkel cell carcinoma.	Phase II. ▪ Locally advanced/mTNBC: IRENE (NCT04445844)
▪ Brand name: Imjudo^®^ ▪ INN: tremelimumab ▪ Target: CTLA4	Advanced/unresectable hepatocellular carcinoma and NSCLC, in combination with durvalumab.	Phase II. ▪ mTNBC PDL1-: NCT03606967
▪ Brand name: Erbitux^®^, generics ▪ INN: cetuximab ▪ Target: EGFR	(Referring to Erbitux^®^) EGFR-expressing/RAS wild-type metastatic colorectal cancer, squamous cell cancer of the head and neck.	Phase Ia/Ib. ▪ Advanced cancer including TNBC: NCT04485013
▪ Brand name: Xgeva^®(I)^/Prolia^®(II)^, generics ▪ INN: denosumab ▪ Target: RANKL	(Referring to ^(I)^ and ^(II)^) ^(I)^ Prevention of SRE in bone metastases, treatment of giant cell tumor of bone|^(II)^ treatment of osteoporosis and bone loss associated with hormone ablation in prostate cancer.	Phase I. ▪ HER2-BC including TNBC: D-BIOMARK (NCT03691311) (pilot)
**(b) Small molecule inhibitor-based targeted therapies.**		
**General information**	**Indication details (EMA)**	**Ongoing TNBC CT at highest phase**
▪ Brand name: Zejula^®^ ▪ INN: niraparib ▪ Target: PARP	Advanced epithelial high-grade ovarian, fallopian tube, or primary peritoneal cancer.	Phase III. ▪ TNBC any BRCA status or HER2-BC with documented deleterious or suspected deleterious tBRCAm: NCT04915755.
▪ Brand name: Lenvima^®^ ▪ INN: lenvatinib ▪ Targets: VEGFR, FGFR, PDGFR	Differentiated thyroid, hepatocellular and endometrial carcinoma.	Phase II. ▪ Solid tumor including mTNBC after 2 therapy lines: NCT03797326. ▪ Recurrent/mTNBC: NCT06140576. ▪ Solid tumors including mTNBC: KEYVIBE-005 (NCT05007106)
▪ Brand name: Fruzaqla^®^/Elunate^®^ ▪ INN: fruquintinib ▪ Target: VEGFR	Metastatic colorectal cancer (mCRC).	Phase II. ▪ Locally advanced/metastatic cancer including TNBC: NCT04577963 NCT05565417 NCT06078670
▪ Brand name: Cometriq^®(I)^/Cabometyx ^®(II)^ ▪ INN: cabozantinib ▪ Target: VEGFR2, c-Met, AKL, RET, others.	**^(I)U^**nresectable locally advanced/metastatic medullary thyroid carcinoma|**^(II)^**renal cell, hepatocellular, differentiated thyroid carcinoma.	Phase II. ▪ Locally advanced/metastatic solid tumors including TNBC: NCT03170960
▪ Brand name: Mektovi^®^ ▪ INN: binimetinib ▪ Target: MEK	Unresectable or metastatic melanoma with a BRAF V600 mutation.	Phase II. ▪ Stage IV or unresectable, recurrent TNBC: InCITe (NCT03971409)
▪ Brand name: Koselugo^®^ ▪ INN: selumetinib ▪ Target: MEK	Symptomatic, inoperable plexiform neurofibromas in pediatric patients ≥ 3 years with neurofibromatosis type 1	Phase II. ▪ mTNBC: NCT03801369 ▪ TNBC: NCT02685657 (unknown status). ▪ Locally advanced/metastatic cancers including TNBC with PI3K/AKT/mTOR or Ras/MEK pathway signaling alterations: TORCMEK (NCT02583542)
▪ Brand name: Mekinist^®^ ▪ INN: trametinib ▪ Target: MEK	Unresectable or metastatic melanoma with a BRAF V600 mutation.	Phase II. ▪ mTNBC: NCT01964924
▪ Brand name: Tarceva^®^, generics ▪ INN: erlotinib ▪ Target: EGFR	(Referring to Tarceva^®^) NSCLC, pancreatic cancer.	None ongoing, completed phase II. ▪ Stage IIIb-IV TNBC: NCT00834678 ▪ mTNBC: NCT00733408
▪ Brand name: Iressa^®^, generics ▪ INN: gefitinib ▪ Target: EGFR	(Referring to Iressa^®^) NSCLC with activating mutations of EGFR-TK.	Phase II. ▪ mTNBC EGFR+: NCT01732276 (unknown status)
▪ Brand name: Xalkori^®^ ▪ INN: crizotinib ▪ Target: ALK, ROS1	ALK+ or ROS1+ NSCLC, ALK+ anaplastic large cell lymphoma, ALK+ inflammatory myofibroblastic tumor.	Phase II. ▪ Lobular breast cancer, diffuse gastric cancer, and TNBC lobular type or CDH1-mutated solid tumors: ROLo (NCT03620643)
▪ Brand name: Nexpovio^®^ ▪ INN: selinexor ▪ Target: *XPO1*	▪ Under additional monitoring. ▪ Indication: multiple myeloma.	Phase I. ▪ Advanced refractory solid tumors, and advanced/mTNBC: START (NCT05035745)
**(c) Other therapies.**		
**General information**	**Indication details (EMA)**	**Ongoing TNBC CT at highest phase**
▪ Brand name: Imlygic^®^ ▪ INN: talimogene laherparepvec (T-VEC) ▪ MoA: oncolytic virus,	Unresectable melanoma	Phase I/II. ▪ TNBC: NCT02779855.
▪ Brand names: Casodex^®^, generics ▪ INN: bicalutamide ▪ MoA: AR antagonist	(Referring to Casodex^®^) Prostate cancer.	Phase II. ▪ TNBC AR+: NCT03090165. ▪ Locally advanced/mTNBC AR+: ABBICAR (NCT06365788)
▪ Brand names: Xtandi^®^ ▪ INN: enzalutamide ▪ MoA: AR antagonist	Prostate cancer.	Phase II. ▪ Early stage TNBC AR+: NCT02750358, ▪ Advanced solid tumors after at least one line of chemotherapy treatment, including TNBC: NCT05252390.

**Table 4:** Onco-immunology therapies in early clinical development stages for TNBC. Ongoing trials refers to the statuses of “not yet recruiting”, “recruiting”, and “active, not recruiting” as stated by August 2024 in the National Library of Medicine USA (NLM) registry Clinicaltrials.gov. Details of the participation criteria in these trials are provided.

**Therapy**	**General Information**	**Ongoing TNBC CT at Highest Phase**
Oncolytic virus	▪ Name(s): pelareorep/Reolysin^®^ ▪ MoA: replicates into cells with activated Ras, induces cytotoxicity.	Phase II. ▪ Locally advanced/mTNBC. IRENE (NCT04445844).
	▪ Name: BT-001 ▪ MoA: intra-tumoral delivery, local expression of GM-CSF and mAb anti-CTLA4 leading to tumor-specific immune response.	Phase I/II. ▪ Advanced/metastatic solid tumors including TNBC: NCT04725331.
Vaccine	▪ Name: not disclosed, “personalized synthetic long peptide vaccine” ▪ Target: neoantigen not disclosed.	Phase II. ▪ mTNBC PDL1-: NCT03606967,
	▪ Several vaccines tested in the same CT: ETBX-011, targeting CEA, adenoviral ETBX-051, targeting brachyury, adenoviral ETBX-061, targeting MUC1, adenoviral GI-4000, targeting mutant Ras proteins, *S. cerevisiae*-derived GI-6207, targeting CEA, *S. cerevisiae*-derived GI-6301, targeting brachyury, *S. cerevisiae*-derived	Phase I/II. ▪ Unresectable/mTNBC: QUILT-3.067 (NCT03387085).
CAR-cell	▪ Name: IVAC_W_bre1_uID and IVAC_M_uID ▪ MoA: mRNA vaccine.	None ongoing, completed phase I ▪ Invasive TNBC: TNBC-MERIT (NCT02316457).
	▪ Name: PVX-410 ▪ MoA: peptidic vaccine.	Phase I. ▪ mTNBC HLA-A2+ (NCT03362060).
	▪ Name: ROR1-CART ▪ MoA: target—ROR1, CART.	None ongoing, terminated phase I. ▪ Advanced ROR1+ malignancies including TNBC: NCT02706392.
	▪ Name: LYL-797 ▪ MoA: target—ROR1, CART.	Phase I. ▪ ROR1+ relapsed/refractory TNBC or NSCLC: NCT05274451.
	▪ Name: CART-meso ▪ MoA: target—mesothelin, CART.	Phase I ▪ Relapsed/refractory advanced malignancies mesothelin+ including TNBC: NCT02580747 (unknown status)
	▪ Name: **TC-510** ▪ MoA: target—mesothelin, CART.	Phase I/II. ▪ Advanced mesothelin+ cancer including TNBC: NCT05451849.
	▪ Name: TnMUC1-CART ▪ MoA: target—TnMUC1, CART.	None ongoing, terminated phase I. ▪ TnMUC1+ advanced cancers, including TNBC: NCT04025216.
	▪ Name: MUC1-CART ▪ MoA: target—MUC1, CART.	Phase I/II. ▪ MUC1+ advanced refractory solid tumors including TNBC: NCT02587689
	▪ Name: MUC1-CAR-pNK ▪ MoA: target—MUC1, CARNK.	Phase I/II. ▪ MUC1+ advanced refractory solid tumors including TNBC: NCT02839954 (unknown status)
	▪ Name: NKG2DL-CARγδT ▪ MoA: target—NKG2DL, CART.	Phase I/II. ▪ Relapsed/refractory solid tumors, including mTNBC. NCT04107142 (unknown status)
	▪ Name: EGFR/B7H3-CART ▪ MoA: targets—EGFR and B7H3 (CD276), CART.	Phase I ▪ EGFR/B7H3+ advanced lung cancer and TNBC: NCT05341492

## Abbreviations

ALK, Anaplastic Lymphoma Kinase; Ang 1/2, Angiopoietin 1/2; ATC, Anatomical Therapeutic Chemical; AR, Androgen Receptor; ASCO, American Society of Clinical Oncology; BET, Bromodomain and Extra-Terminal motif; BRCA, BReast Cancer; BRD, Bromodomain-containing protein; CAR, Chimeric Antigen Receptor; CEA, CarcinoEmbryonic Antigen; CDH1: Cadherin 1 (or E-Cadherin); CDK, Cyclin-Dependent Kinase; CT, clinical trials; CTLA4, Cytotoxic T-Lymphocyte Associated protein 4; EGFR, Epidermic Growth Factor Receptor; EMA, European Medicines Agency; ER, Estrogen Receptor; ErbB, Erythroblastic leukemia viral oncogene homologue; ESMO, European Society for Medical Oncology; FDA, Food and Drug Administration (USA); FGFR, Fibroblast Growth Factor Receptor; GEJ, gastroesophageal junction; GM-CSF, Granulocyte-Macrophage Colony-Stimulating Factor; HLA, Human Leukocyte Antigen; HER2, Human epidermal growth factor receptor 2; HMGA2, High Mobility Group AT-Hook 2; HR, Hormone Receptor; ICI, immune checkpoint inhibitor; INN, International Nonproprietary Name; ILA1RAP, Interleukin-1 receptor accessory protein; MA, Marketing Authorization; mAb, monoclonal antibody; MAPK, Mitogen-Activated Protein Kinase (several synonyms as Extracellular signal-Regulated Kinase [ERK]); MoA, Mechanism of Action; MMAE, monomethyl auristatin E; MMAF, monomethyl auristatin E; MSI-H/dMMR, High levels of MicroSatellite Instability (MSI-H)/deficient MisMatch Repair (dMMR); MUC1: Mucin short variant S1; mTOR, mammalian Target Of Rapamycin; NMPA, National Medical Products Administration (China); NCCN, National Comprehensive Cancer Network; NK, natural killer (haNK, human allogenic NK; pNK, placental-derived NK); NKG2D, Natural Killer Group 2D also known as Killer cell lectin like receptor K1 (KLRK1) or CD314; NSCLC, Non-Small Cell Lung Cancer; PARP, Poly ADP Ribose Polymerase; PD1, Programmed Cell Death Protein 1 or CD279; PDGFR, Platelet-Derived Growth Factor Receptor; PDL1, Programmed Cell Death Ligand 1 or CD274; PDx, Patient-Derived xenograft; PI3K, Phosphatidylinositol 3-Kinase; PR, Progesterone Receptor; RANK, Receptor Activator of NFƙB; ROR1, receptor tyrosine kinase like orphan receptor 1; RT, radiotherapy; SCLC, Small Cell Lung Cancer; SINE, Selective Inhibitor of Nuclear Export; SRE, skeletal related events; TGFβRII, TGF β receptor type 2; TIGIT, T cell immunoglobulin and ITIM domain; TIL, Tumor-Infiltrating Lymphocyte; TKI: Tyrosine Kinase Inhibitor; Trop2, Trophoblast cell-surface antigen 2; VEGF, Vascular Endothelial Growth Factor; VEGFR: VEGF Receptor; XPO1, Exportin 1; ZIP6, Zinc-regulated, Iron-regulated transporter-like Protein 6.
